# Toxicological study of the butanol fractionated root extract of *Asparagus africanus* Lam., on some blood parameter and histopathology of liver and kidney in mice

**DOI:** 10.1186/s13104-016-1861-5

**Published:** 2016-01-27

**Authors:** Sintayehu Kebede, Mekbeb Afework, Asfaw Debella, Wondwossen Ergete, Eyasu Makonnen

**Affiliations:** Department of Anatomy, Faculty of Medicine, Addis Ababa University, P.O. Box 18309, Addis Ababa, Ethiopia; Department of Anatomy,Medical Faculty, Addis Ababa University, Addis Ababa, Ethiopia; Ethiopian Public Health Institute, Traditional and Modern Medicine Drug Research Directorate, Addis Ababa, Ethiopia; Department of Pathology, Medical Faculty, Addis Ababa University, Addis Ababa, Ethiopia; Department of Pharmacology, Medical Faculty, Addis Ababa University, Addis Ababa, Ethiopia

**Keywords:** *Asparagus africanus*, Butanol fraction, Blood parameters, Histopathology

## Abstract

**Background:**

The butanol fractionated root extract of *Asparagus africanus* Lam., a traditional herb widely used to treat various ailments were analyzed for the presence of potential toxicity after single (acute) and repeated (subchronic) dose oral administration in adult swiss albino mice using gavages.

**Methods:**

For the acute study, butanol fractionated extract of the plant was administered in single doses of 1000, 3000 and 5000 mg/kg body weight. In the sub-chronic dose study, the extract was administered at doses of 300 and 600 mg/kg body weight/day for 42 days. Selected hematological and biochemical parameters of the blood followed by histopathological analysis were investigated after 42 days of daily administrations. The results were expressed as M ± SE, and differences at P < 0.05 was considered significant. One way analysis of variance (ANOVA) and least significant difference tests were employed to check the significant differences between the various parameters of the experimental groups.

**Results:**

In the acute study, the extract did not caused dose-dependent general behavioral adverse effects, body weight change and mortality. The single dose toxicity studies therefore showed that the butanol fraction of the extract has high safety profile when given orally. After 42 days of daily dosing, in the sub-chronic study, no clinically significant changes were observed for hematological and biochemical parameters. Except an occasional small number of focal mononuclear lymphocytic cells infiltrations around the central and portal triad of the liver of a few mice, the histopathological parameters do not show significant change.

**Conclusion:**

It is concluded that, the butanol fractionated extract from *A. africanus* at the given dose does not show significant toxicity. The presence of focal inflammation on the liver of a few mice may be associated to the presence of flavonoid glycoside in the butanol fractionated extract.

## Background

Many plants have shown very effective medicinal values for various ailments of human and domestic animals. Studies have witnessed several of the pharmaceuticals currently available to physicians have a long history of use as herbal remedies. Digoxin and digitoxin, drug for heart failure and atrial dysrhythmias, from digitalis leaves [27]; quinine, antimalarial drug, from cinchona bark [19] and anti-cancer compound bruceatin, from the ethiopian plant, *Brucea antidysentrica* [14], are examples of the contributions of traditional pharmacopoeia.

Despite its being natural, reputation and continued use over many centuries, recent studies on laboratory animals have shown many plants used as medicinal activity have potential toxicity on blood parameters and histopathology of internal organs [5, 7, 18]. In addition, the advancement of technology has enabled to detect minute amounts of carcinogenic and toxic chemicals and recognize potentially hazardous effects of some of the herbs used in traditional medicines [25].

It therefore appears that, although traditional medicine is widely used to treat various diseases and often more available and affordable than modern medicine, it is not without limitations. *Asparagus africanus* Lam., commonly known with its vernacular name “Seriti” (Afaan Oromo) is one of the most effective medicinal plants widely used to treat malaria [10], impotency [29], diarrhea [1], lishmaniansis, bilharziasis, syphilis and gonorrhoea [26] and fertility [15]. The plant belongs to family *Asparagaceae* which includes 300 species in the genus Asparagus, widely distributed throughout Africa. It is a perennial shrub or climber with stems up to 6 m high growing between 700 and 3800 m above sea level [11, 17]. Different studies have shown antimalarial activity of the plant with different potential of parasitimal suppression. The crude extracts of *A. africanus* for instant have shown a parasitimial suppression of 46.1 % (root parts) and 40.7 % (aerial parts) on Swiss albino mice [12]. The methanol extracts of the plant found to have parasitaemia suppression against *Plasomodium berghei* at a dose of 200 mg/kg [12]. Butanol fractionated extract of *A. africanus* showed the highest inhibition (85.94 %) of *P. berghei* parasitaemia in the swiss albino mice [10]. In spite of such widespread use of the plant, its safety based on their effective dose is not yet known.

With the problems of increasing drug resistance and difficulties in getting affordable effective antimalarial drugs, traditional medicines, mainly of plant sources could be an important and sustainable ways of treatment if supported by clinical evidences of safety and efficacy. Medicinal plants not only complement modern medicine but also are the basis for the development of modern pharmaceuticals. Despite highest (85.94 %) inhibition of *P. bergei* parasitaemia, no clinical evidence of safety (toxicity) yet found on butanol fractionated extract of *A. africanus*. This study is therefore aimed to investigate the toxicity of butanol fractionated extract of *A. africanus* Lam., on some blood parameters and histopathology of liver and kidney in mice.

## Methods

### Plant material collection and processing

The fresh root of *A. africanus* were collected from the border of Shashamane and Awassa towns about 270 km south of Addis Ababa. The plant was identified by a taxonomist in the Traditional and Modern Medicine Drug Research Directorate (formerly called Ethiopian Public Health Institute) at the Ethiopian Public Health Institute (formerly called Ethiopian Health and Nutrition Research Institute). A sample of the plant was deposited at the Herbarium of Traditional and Modern Medicine Drug Research Directorate with Voucher Specimen number Herb No. AA-2163. The collected plant roots were cut into pieces, cleaned from extraneous materials and then dried at ambient temperature (23–26 °C) and grounded to powder using a sample mill.

### Hydro-alcoholic extraction of the plant material

In this study both the hydro-alcoholic extract and solvent fractionation of the plant material were done as the method employed on efficacy study by Debebe [10]. Briefly, hydro-alcoholic extract yields were obtained by soaking 400 g of plant powder in 3 L of 70 % ethanol to fully immerse and wet the plant powder. These were placed on orbital shaker rotating 120 rpm for 48 h for regular infusion. On the 3rd day, the mixture was left to settle and the clear decanted supernatant was filtered using Whatman filter paper No. 3, 18.5 cm, England into a clean container. The extract was then reduced to dryness under low pressure using a rotary vacuum evaporator (BUCHI type R-205, with Aspirator pump, Switzerland) at a temperature of 40 °C rotating 40 rpm. Finally the filtrate in a container was put in a water bath (BUCHI Heating Bath B-490) to remove all the alcohol left in the extract. The crude extract was then stored in labeled sterile glass vials at −20 °C until used.

### Solvent–solvent fractionation of plant material

The butanol fractions were obtained after the hydro-alcoholic extract were exhaustively defatted respectively with n-hexane (6 repeat) and chloroform (8 repeat) respectively. The aqueous residue after de-fat was further partitioned with 50 ml of n-butanol for eight exhaustive separate and successive mixing and filtrations steps. The n-butanol fractionates were then combined, concentrated and labeled as butanol fraction (BF). The weight in the dry extract was expressed as total mass of the powder.

### Experimental animal preparation

The study was conducted on Swiss albino mice (weighing 20–25 g) obtained from colonies in the animal unit of the Ethiopian Public Health Institute. The animals were acclimatized to laboratory conditions in the Traditional and Modern Medicine Drug Research Directorate for 1 week prior to the experimental procedure to minimize any nonspecific stress. After a week, body weight of each mouse was measured in gram and recorded as the initial body weight. The mice were housed in standard aluminum cages maintained at normal laboratory conditions of temperature (21 ± 2 °C), relative humidity of 65 ± 0.5 % and 12 h light–dark cycle with free access to standard commercial diets and clean tap water *ad*-*labium* until the end of the experiment based on WHO Annex II Research guidelines for evaluating the safety and efficacy of herbal medicines [30].

### Ethical clearance

Ethical approval for the animal used for this study was obtained from Addis Ababa University Medical Faculty Institutional Review Board Protocol number: 158/09/Anat.

### Single dose toxicity study

The single dose study was carried out in 24 adult female mice randomly distributed into four groups (group I, II, III and IV), each with six mice. The plant extract was orally administered to the mice in groups I, II and III respectively, in a single dose of 1000, 3000 and 5000 mg/kg body weight, while distilled water was administered to control group (group IV) using a stomach tube gavages based on WHO Annex II; research guidelines for evaluating the safety and efficacy of herbal medicines [30]. Changes like muscular weakness, behavior change, salivation, diarrhea, body weight and food intake in comparison to the controls, were recorded separately for each animal for 14 days.

### Repeated dose toxicity study

#### Selection of dose and duration of dosing

Effective dose for parasitimal suppression 300 mg/kg body weight [10] and its double (i.e., 600 mg/kg body weight) were selected for repeated dose toxicological investigation of the plant extract as the appropriate doses for this particular study for 42 days [30].

#### Administration of the extract

For repeated dose toxicity study, 36 mice were randomly distributed into three groups, each with 12 mice and were assigned as group I, II and III. group I and II were respectively treated with 300 and 600 mg/kg body weight, while mice in group III were treated with vehicle (distilled water) [30]. Administrations of the extract as well as the vehicle into the mice were done orally by using stomach tube gavages [30].

#### Hematological and biochemical parameters and histopathology of liver and kidney

After 24 h of the administration of the extract for the last time, i.e., at the end of the 6th week, the animals were anaesthetized under diethyl ether and blood sample for bioassays was immediately collected from each mouse by a cardiac puncture using sterile needle and syringe for evaluation of any change in hematological and biochemical parameters [30]. Following collection of blood sample, tissue section from liver and kidney of all mice in each groups were immediately removed and processed for section preparation for light microscopy (fixed, dehydrated, cleared, impregnated, sectioned and placed in microscopic slides) and stained with Hrris hematoxylin and counterstained in 1 % alcoholic eosin for microscopic slides. At the end of every week, change in body weight was analyzed by recording the body weight of each animal.

### Statistical analysis

All data were packed and analyzed by SPSS statistical Microsoftware. Mean values (M) and standard errors (SE) of the parameters were calculated and the results were expressed as M ± SE. One way analysis of variance (ANOVA) and least significant difference tests were employed to check the significant differences between the various parameters of the experimental groups. In addition, Dunnett t-tests which treat one group as a control, and compare all other groups against it was employed to compare the values within a parameter and to evaluate the relationship between the variables. Differences at P < 0.05 was considered significant.

## Results

### Effect of single dose treatment of the butanol fractionated extract of *Asparagus africanus*

Following a single treatment with fractionated extract of *A. africanus* at doses of 1000, 3000 and 5000 mg/kg body weight, no signs and symptoms of toxicity on behavior nor death were observed throughout the 14 days of the study period. Comparison of body growth patterns and body weight gains between the test groups and the control showed no significant differences as shown in Table 1.

**Table 1:** Comparison of body weight change among butanol fractionated extract of *A. africanus* treated groups, at doses of 1000, 3000 and 5000 mg/kg body weight and control mice during the 14 days of observation

Group	Dose (mg/kg)	Initial body weight	Body weight at day 7	Final body weight at day 14
III	1000	20.84 ± 0.80	27.62 ± 0.40	30.46 ± 0.45
II	3000	20.83 ± 0.35	27.57 ± 0.70	30.13 ± 0.48
I	5000	21.43 ± 0.54	27.94 ± 0.71	30.90 ± 0.69
IV	Control	20.66 ± 0.44	26.90 ± 0.68	30.07 ± 0.45

Data are expressed as mean ± SEM, n = 6/group

### Effect of repeated dose treatment of the fraction of *Asparagus africanus*

#### Effects of the fraction on hematological parameters of blood

No statistical significant (at P > 0.05) change was observed in the hematological parameters of the mice treated with the repeated doses of 300 and 600 mg/kg body weight/day of the fractionated extracts of *A. africanus* as compared to the controls as shown in Table 2. However, a non-significant increase in WBC in both groups, decrease in platelet count in mice treated with 600 mg/kg and increase in mice treated with 300 mg/kg were seen as compared to the control.

**Table 2:** Comparison of hematological parameters among fractionated extract of *A. africanus* treated groups at doses of 300 and 600 mg/kg body weight/day, and the control mice

Hematological parameter	Control	300 mg/kg bwt			600 mg/kg bwt		
		Values	% of mean difference	*P* value	Values	% of mean difference	P value
RBC (×10^6^/µL)	10.93 ± 0.38	11.48 ± 0.46	4.79	0.596	11.33 ± 0.64	3.55	0.468
Hemoglobin (g/dL)	15.63 ± 0.75	16.47 ± 0.58	5.10	0.383	16.57 ± 0.75	5.67	0.433
Hematocrit (%)	56.97 ± 1.72	60.20 ± 2.29	5.37	0.756	56.05 ± 1.91	−1.61	0.294
MCV (fL)	52.17 ± 0.63	52.47 ± 1.27	0.57	0.662	52.83 ± 1.07	1.25	0.843
MCH (pg)	14.30 ± 0.21	14.33 ± 0.29	0.21	0.309	14.70 ± 0.27	2.72	0.929
MCHC (g/dL)	27.40 ± 0.59	27.37 ± 0.15	−0.11	0.593	27.80 ± 0.63	1.44	0.964
PLAT (10^3^/µL)	581.00 ± 38.2	702.67 ± 99.3	35.56	0.564	455.33 ± 288.7	−21.67	0.169
WBC (10^3^/µL)	4.76 ± 1.42	6.76 ± 2.36	29.59	0.352	7.97 ± 2.76	40.28	0.553

Data are expressed as mean ± SEM, n = 12

*Bwt* body weight

#### Effects of the fraction on biochemical parameters of the blood

Values of the functional tests for liver (AST, ALP and ALT) and kidney (urea and creatinine) in the extract treated mice as compared to those of the controls are shown in Table 3. All these values of the functional test showed no significant change.

**Table 3:** Comparison of biochemical parameters among fractionated extract of *A. africanus* treated groups, at doses of 300 and 600 mg/kg body weight/day, and the control mice

Biochemical parameters	Control	300 mg/kg bwt			600 mg/kg bwt		
		Value	% of mean difference	P value	Value	% of mean difference	P value
AST (IU/L)	208.17 ± 44.84	208.17 ± 51.63	0	1.00	261.20 ± 32.38	17.06	0.49
ALP (IU/L)	74.00 ± 29.46	75.00 ± 10.25	1.33	0.34	76.32 ± 31.45	3.03	0.25
ALT (IU/L)	107.50 ± 34.88	84.67 ± 9.64	−21.24	0.47	82.80 ± 13.41	−25	0.40
Urea (mg/dL)	49.00 ± 2.91	52.33 ± 6.92	6.36	0.60	46.40 ± 1.33	−6.47	0.62
Creatnine (mg/dL)	0.88 ± 0.11	0.83 ± 0.11	−5.68	0.75	1.24 ± 0.16	26.67	0.08

Data are expressed as Mean ± SEM, n = 12

#### Effect of the fraction on behavior and body weight

Daily cage-side observations did not reveal any physical changes in the skin, fur, eyes, and respiratory system and general behavioral patterns. No autonomic effect such as salivation, diarrhea and urination were observed throughout the 42 days of administration period as compared to the controls. All of the treated as well as control mice survived until the scheduled necropsy. Macroscopic autopsy examination also did not reveal any pathological findings in the liver and kidneys. Comparable increase in body weight gains were observed for all the extract as well as the control mice as shown in Table 4.

**Table 4:** Comparison of body weight change among fractionated extract of *A. africanus* treated groups, at doses of 300 and 600 mg/kg body weight/day, and the control mice

Group	Week 0 bwt (g)	Week 1 bwt (g)	Week 2 bwt (g)	Week 3 bwt (g)	Week 4 bwt (g)	Week 5 bwt (g)	Week 6 bwt (g)
G1	135.23 ± 2.31	144.18 ± 8.76	148.79 ± 11.79	156.28 ± 14.88	158.05 ± 32.20	154.93 ± 27.53	179.73 ± 16.17
G2	135.82 ± 1.37	158.97 ± 16.62	161.23 ± 16.12	162.81 ± 13.54	176.86 ± 15.29	173.11 ± 8.67	184.93 ± 13.33
G3	135.63 ± 3.52	162.29 ± 16.81	166.42 ± 18.99	171.05 ± 20.35	180.33 ± 27.41	180.01 ± 23.02	193.46 ± 21.56

Values are expressed as mean ± SEM; n = 12

#### Effect of the fraction on the histology of the liver

**Fig. 1:**
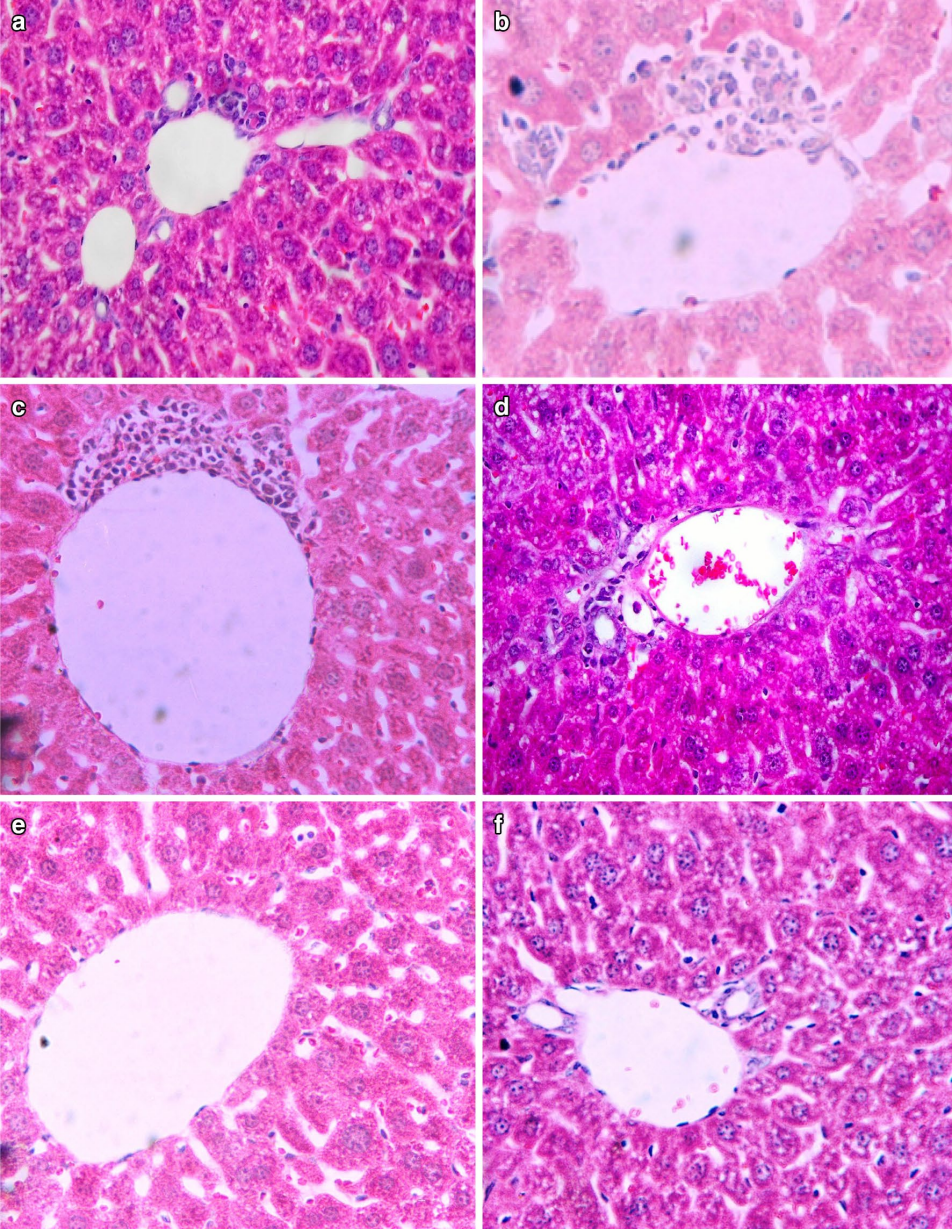
Photomicrograph of section of mice Liver after 42 days of treatment with butanol fractionated extract of *A. africanus* at 600 mg/kg body weight/day (**a**, **c**) and 300 mg/kg body weight/day (**b**, **d**) as compared to the control (**e**, **f**). Peri-portal vein focal mononuclear lymphocytic cell infiltration around both portal and central veins of the treated mice, and those infiltrations are more around the central veins (sections were stained with H&E, ×2000)

Microscopic examination of tissue sections of liver under light microscope from mice treated with the butanol fractionated extract of *A. africanus* at 300 and 600 mg/kg body weight/day doses showed no treatment related pathological findings and were comparable to those of the controls. However, there were occasional focal mononuclear lymphocytic cellular infiltration around the portal and central vein of few slides as shown in Fig. 1a–d.

#### Effect of the fraction on histology of the kidney

**Fig. 2:**
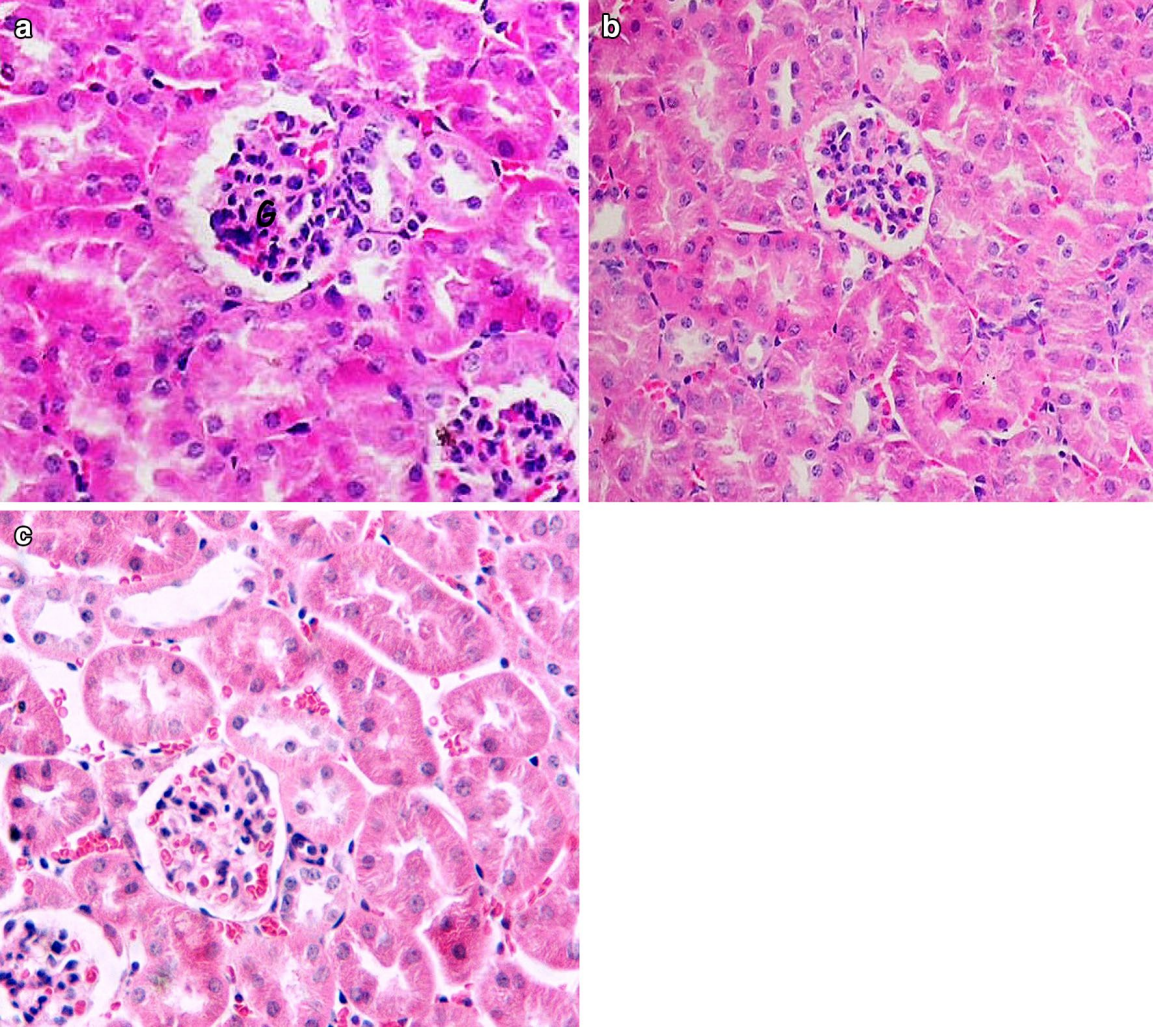
Photomicrographs of section of the mice kidney of 600 mg/kg body weight/day dose extract treated mice (**a**) and 300 mg/kg body weight/day (**b**), as compared to the vehicle treated control mice (**c**). Sections were stained with H&E, ×2000 for **a**–**c**

Microscopic examinations of tissue sections of the kidney from the butanol fractionated extract treated mice at 600 and 300 mg/kg body weight/day doses showed normal histology with no evident of treatment related changes as compared to those of controls Fig. 2.

## Discussion

In this study, to determine the LD50, the butanol fractionated extract with a minimum dose of 1000 and a maximum dose of 3000 mg/kg body weight were first tried in a single administration. As recommended by WHO guide line for dose selection, the dose was then raised to 5000 mg/kg body weight. In all the three doses, the mice treated with extract did not show change in food consumption, behavioral patterns, body temperature, and respiratory rate. No autonomic effects such as salivation, diarrhea and urination were seen. There were comparable body weight gains among the extract treated mice as compared to those of the controls. All the treated mice survived until the scheduled necropsy. This finding therefore, suggested that the LD50 of the fractionated extract appears to be greater than 5000 mg/kg body weight. Hence, the single dose administration of the extract has high safety profile when given orally.

Analysis of red blood cell indices (Table 2) showed no significant reduction following repeated administration of the butanol fractionated extract of *A. africanus*. This result indicates neither lysis nor inhibition in blood cells synthesis by any of the active constituents that may exist in fractionated extract of *A. africanus*. The unlikelihood of the extract to induce anemia even after long use on red blood cell indices may be due to the inability of the extract to distract the matured red blood cell and inhibit erythropoiesis. This is in agreement with other related studies like the long term treatment with *Teucrium polium* total extract and its failure to induce any toxic effect on the matured red blood cells and hence was suggested to induce no anemia [16]. On the other hand, chronic treatments with plants extract like *S. lycopersicum*, *M. perennis*, *M. annua*, *E. balsamifera*, *E. hirta*, *E. heterophylla*, *E. lateriflora*, *E. hyssopifolia* and *Cassia italica* are known to cause destruction of hematological parameters leading to anemia in animals [3, 4, 20].

Leukocytes are known to increase sharply as the first line defense of the body or in response to toxic environment when infection occurs. The absence of significant increase in the WBC after 42 days of chronic treatment with the extract suggests for lack of damaging toxic effect of the plant extract on the mice. The non-significant increases of the treated mice at P < 0.05 observed in the WBC count by 29.59 % and 40.28 %, respectively for 300 and 600 mg/kg/body weight doses as compared to the control may probably be due to the normal responses of the mice to foreign bodies or stress associated with the sub-chronic toxicity of the extract. It is known that chronic treatment with some plant extract cause a non significant increase in WBC parameter as a normal response to the extract [2, 21].

Significant elevation of platelets (thrombocytosis) could predispose to hypercoagulable state, which results to spontaneous intravascular clotting and thromboembolism that may lead to stroke and heart attack [6, 7]. On the other hand, significant decrease in platelets (thrombocytopaenia) could increase the tendency to bleed and have anticoagulant property [9]. In this study, the observation that platelets count was not affected significantly at any of the dose employed suggests that the fraction does not interfere with coagulation.

Generally, following exposure to a toxic substance, a significant reduction in body weight by more than 10 % from the initial body weight is a simple and sensitive index of toxicity [28]. In this study administration of the extract of *A. africanus* for 42 days at both of the investigated doses, apart from slight variation, did not produce significant change in the body weight as compared to the controls. The slight non-significant differences observed may have resulted from physiological variations in the mice such as food intake and metabolism as suggested in other related study [22]. The lack of adverse effect of the plant on body weight is thus a pointer to the safety of prolonged oral administration of the butanol fractionated extract of *A. africanus*.

After treatment of the butanol fractionated extract at doses of 600 mg/kg body weight there were trends of increment in AST by 17.06 % and decrement in ALT by 25 % as compared to the control. However, as these were not statistically significant such changes appear not to be extract related. In a comparable study after administration of *Glinus lotoides* and *Hagenia abyssinica* the level of ALT decreased and the level of AST increased non-significantly and the author concluded that the extract is not hepatotoxic [23]. Moreover, after sub chronic treatment with butanol fractionated extract of *A. Africanus* at both doses the level of ALP did not change significantly as compared to the control suggesting the extract is not toxic to the liver or damaging to the integrity of the bile duct.

Investigation for the toxic potential of a chemical is incomplete without gross and histopathological evaluation [8]. Following exposure to a toxic substance, a significant reduction in body weight by more than 10 % from the initial body weight is a simple and sensitive index of toxicity [28]. The non significant differences between the weight of mice in the experimental and control group observed in this study may have resulted from physiological variations in the mice such as food intake and metabolism as suggested in other related study [22].

Kidneys are particularly vulnerable to toxic agents given their high rate of perfusion by blood and their ability to concentrate a range of substances in the tubular lumen. Following administration of the butanol fractionated extract, a non-significant increase by 5.68 and decrease by 26.67 % of kidney biochemical marker creatinine was seen in mice treated respectively with 300 and 600 mg/kg body weight/day. Furthermore, the urea level increased non significantly by 6.37 % for mice treated with 300 mg/kg and decreased non significant by 6.47 % for mice treated with 600 mg/kg as compared to the control group. The current finding may suggest that the activity of protein metabolism was maintained within the normal range due to relatively non-toxic effect of the extract. Similar suggestion was made after non significant changes in creatinine and urea levels were seen following repeated administration of *Sphenocentrum jollyanum* extract [24]. Hence the present result probably suggests for absence of any functional damage to the kidney showing the extract did not interfere with the renal capacity to excrete the metabolites.

In the histo-pathological analysis of liver, apart from an occasional small number of focal mononuclear lymphocytic cells infiltration around the central and portal triad no gross or microscopic lesions were found following the 42 days of administration of the extract at 300 and 600 mg/kg body weight/day doses as compared to the controls. Hence, in this study the extract from *A. Africanus* appears to be not nephrotoxic at 300 and 600 mg/kg body weight/day doses studied as they did not show any gross or microscopic lesions. The focal mononuclear lymphocytic cell infiltration observed may be because of the flavonoid glycoside present in the *A. africanus.* However, this should be ascertained by phytochemical screening of *A. africanus*. The presence of glycosides on the other hand has been suggested to cause focal inflammation around the portal triad on the liver with no effect on the kidney [13].

The hematological and biochemical parameters of mice treated with the extract in repeated dose toxicity study showed no significant change as compared to the control mice. Lack of such statistically significant effect may be due to the absences of toxic secondary metabolites in the butanol fractionated extract of *A. africanus*. The non-significant change in biochemical parameters on the other hand tells no tissue damage due to the administration of the extract at 300 and 600 mg/kg/body/weight doses in mice. Indeed, such observation in the biochemical parameter is in agreement with the histopathological findings in which no dose related tissue damage appeared, except for the presence of focal mononuclear lymphocytic cell infiltrations around the central vein and portal triad of few mice.

## Conclusion

The present study revealed that the butanol fractionated extract from the root of *A. Africanus* is not hepato-nephro- and hemato-toxic at 300 and 600 mg/kg body weight/day as shown from body weight, biochemical, hematological and histopathological findings. As found from the present study *A. Africanus* is non-toxic at the studied dose in mice and this may call for further investigations including phytochemical screening as well as trial in other animals towards the development of drug from *A. Africanus* for its claimed traditional therapeutic values.

## Abbreviations

AAU: Addis Ababa University
ALP: alkaline phosphatase
ALT: alanin aminotransferase
AST: asparate aminotransferase
CNS: central nervous system
D.P.X: dibutyl phthalate in xylene
dL: deciliter
EDTA: ethylene diamine tetraacetic acid
fL: femtoliter
LD_50_: lethal dose that kills half of the animals
MCH: mean corpuscular hemoglobin
MF: medical faculty
MCV: mean corpuscular volume
MCHC: mean corpuscular hemoglobin concentration
Pg: pictogram
RBC: red blood cell
SEM: standard error of mean
SPSS: statistical package for social science
WBC: white blood cell
WHO: World Health Organization
µL: microliter
